# Juvenile stress induces behavioral change and affects perineuronal net formation in juvenile mice

**DOI:** 10.1186/s12868-018-0442-z

**Published:** 2018-07-16

**Authors:** Hiroshi Ueno, Shunsuke Suemitsu, Shinji Murakami, Naoya Kitamura, Kenta Wani, Yosuke Matsumoto, Motoi Okamoto, Shozo Aoki, Takeshi Ishihara

**Affiliations:** 1 Department of Medical Technology, Kawasaki University of Medical Welfare, 288, Matsushima, Kurashiki, Okayama 701-0193 Japan; 2 Department of Medical Technology, Graduate School of Health Sciences, Okayama University, Okayama, 700-8558 Japan; 3 Department of Psychiatry, Kawasaki Medical School, Kurashiki, 701-0192 Japan; 4 Department of Neuropsychiatry, Graduate School of Medicine, Dentistry and Pharmaceutical Sciences, Okayama University, Okayama, 700-8558 Japan

**Keywords:** Behavior, Juvenile, Mouse, Perineuronal nets, Parvalbumin, Stress

## Abstract

**Background:**

Many neuropsychiatric disorders develop in early life. Although the mechanisms involved have not been elucidated, it is possible that functional abnormalities of parvalbumin-positive interneurons (PV neurons) are present. Several previous studies have shown that juvenile stress is implicated in the development of neuropsychiatric disorders. We aimed to clarify the effects of juvenile stress on behavior and on the central nervous system. We investigated behavioral abnormalities of chronically-stressed mice during juvenilehood and the effect of juvenile stress on PV neurons and WFA-positive perineuronal nets (PNNs), which are associated with vulnerability and plasticity in the mouse brain.

**Results:**

Due to juvenile stress, mice showed neurodevelopmental disorder-like behavior. Juvenile stressed mice did not show depressive-like behaviors, but on the contrary, they showed increased activity and decreased anxiety-like behavior. In the central nervous system of juvenile stressed mice, the fluorescence intensity of WFA-positive PNNs decreased, which may signify increased vulnerability.

**Conclusion:**

This study suggested that juvenile stressed mice showed behavioral abnormalities, resembling those seen in neuropsychiatric disorders, and increased brain vulnerability.

## Background

Many neuropsychiatric disorders are diagnosed at puberty [1]. Approximately half of adult neuropsychiatric disorders begin in adolescence [2, 3]. However, the cause of the onset remains unclear. It has been reported that the onset of neuropsychiatric disorders such as anxiety, neurosis, depression, post-traumatic stress disorder (PTSD), and schizophrenia are associated with stress exposure during juvenilehood [4, 5]. In recent years, many children worldwide are experiencing stress [6]. Chronic stress is known as a major risk factor for the onset of numerous neuropsychiatric disorders including depression [7–9].

Human behavior is greatly affected by the environment both during childhood and adolescence [10–12]. The maturing brain is very sensitive to stress [13–15]. At this period, stressful events are related to later social and emotional maladjusted behaviors [16]. In animal experiment models, animals stressed in early childhood show increased anxiety-like behavior [17], decreased spatial memory [18], increased corticosterone secretion [19], and altered hippocampal size after maturation [18, 20].

Many common early-stage stress experiment models have focused on the period during lactation through maternal deprivation and separation. However, the development of the pup brain continues after weaning, and brain development is affected by environmental factors. In this study, we focused on mice after weaning. At this time, mice already act independently, and some areas of the central nervous system have matured, but many other brain areas have not [21–23]. Mice at postnatal week 4 are considered to be in the state before human juvenilehood, and their brain is still in the developmental stage [14]. Although the mechanisms involved in stress-related neuropsychiatric disorder development during juvenilehood and adolescence have not been elucidated, it seems that environment, physiology, and heredity are all implicated in a complicatedly interrelated manner [24]. Using animal experiment models, we are just beginning to understand how juvenile organs react to stress [25].

It has been suggested that functional abnormalities in parvalbumin-positive interneurons (PV neurons) are one cause of anxiety, neurosis, depression, and schizophrenia [26–32]. PV neurons are GABAergic interneurons [33–35]. In the central nervous system, GABAergic interneurons mature after birth, and abnormalities in GABAergic interneurons have been reported in numerous neuropsychiatric disorders [36–40]. PV neurons mature depending on environmental inputs around juvenilehood [41–43]. PV neurons form inhibitory synapses at the cell body and axon initial segments of pyramidal cells, and regulate the synchronous firing of pyramidal cells [44, 45]. Dysfunction of PV neurons causes mental disease-like behavior in mice [46, 47].

After birth, the cell bodies, proximal dendrites, and axon initial segments of many PV neurons are covered with special extracellular matrix molecules [48, 49]. This extracellular matrix molecules are called the perineuronal net (PNN). The PNN consists of hyaluronic acid, link proteins, tenascin, and aggrecan, versican, brevican, and neurocan, which are lecticans belonging to the family of chondroitin sulfate proteoglycans [50–53]. Lectin *Wisteria floribunda* agglutinin (WFA), which binds to *N*-acetylgalactosamine residues, is widely used to visualize PNNs [54]. Although the function of PNNs has not been clarified, it has been shown that they exert neuroplasticity control and have neuroprotective effects [55]. It is thought that the critical period ends by the formation of PNNs around PV neurons [56, 57]. Loss of PNNs around PV neurons restores plasticity, and reduces the excitability of PV neurons [58–60]. PNNs also protect PV neurons from oxidative stress [61, 62]. Altered PNNs have been reported postmortem in the brains of patients with schizophrenia and depression [63, 64], and mice with PNN dysfunction show behavioral abnormalities, such as those seen in neuropsychiatric disorders [65–67].

Behavioral abnormalities after maturation due to early life stress have been examined in detail, but behavioral anomalies in juvenilehoods under stress have not been clearly defined. If functional and structural anomalies are maintained until maturity, it is necessary to diagnose young people who are experiencing stress as soon as possible. Therefore, we aimed to clarify behavioral abnormalities in mice that had experienced stress during juvenilehood. In addition, we investigated the influence of stress on both PV neurons and PNNs in each brain region (frontal cortex, motor cortex, and the hippocampus) in juvenile mice.

The PV immunostaining-delineated CA2 neurons have not distinguishable differences in cell morphology compared with CA1 and CA3 regions. Hippocampal area CA2 is excluded from present study. Studies of neuropsychiatric disorders have implicated PV neuronal abnormalities in region-specific dysfunction and not in the sensory cortex. These reports indicate that there is region-specific vulnerability of PV neurons to neuropsychiatric conditions in the cortex. The motor cortex on the same section as the frontal cortex was examined simultaneously.

We physically and socially stressed mice for 10 days, which were at different developmental periods: early childhood (from postnatal day 21–30) and maturation phase (from postnatal day 81–90) on the same stress schedule. Therefore, in this study, we aimed to clarify the influence of stress on juvenile behaviors and on the formation of developing PV neurons and PNNs.

## Methods

### Animals

**Fig. 1:**
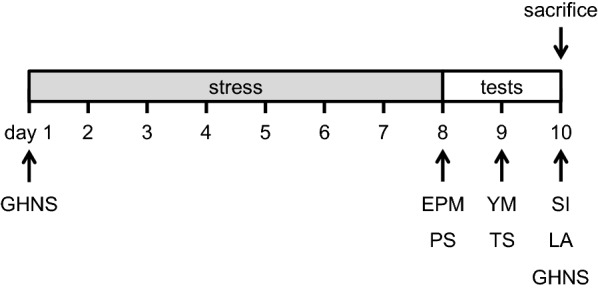
Experimental time schedule. Animals in the stress groups were subjected to stress once a day from P21 (juvenile) or P71 (adult). Animals were subjected to two behavioral tests per day. *EPM* elevated plus maze, *PS* Porsolt forced swim, *YM* Y-maze, *TS* tail-suspension, *SI* social interaction, *LA* locomotor activity, *GHNS* general health and neurological screening

All animal experiments were performed in accordance with the U.S. National Institutes of Health (NIH) Guide for the Care and Use of Laboratory Animals (NIH Publication No. 80-23, revised in 1996) and were approved by the Committee for Animal Experiments at Kawasaki Medical School Advanced Research Center. All efforts were made to minimize the number of animals used and their suffering. The day of birth was designated as postnatal day (P0). Animals were purchased from Charles River Laboratories (Kanagawa, Japan) and housed in cages (5 animals/cage) with food and water provided ad libitum under a 12 h light/dark cycle at 23–26 °C. We used C57BL/6N male mice aged P21 (juvenile) and P71 (adult). Mice between the age of P0 and P28 are termed juvenile and mice between P28 and P56 should be termed adolescence. Since the adolescent period is quite ambiguity in rodents, we chose to start stress during what is considered to be the juvenile period (P21–30). Adult mice were exposed to stress from P71 to P80. The animals were randomly assigned to either the control (n = 10) or stress groups (n = 10). All behavioral tests were conducted in behavioral testing rooms between 08.00 and 18.00 h during the light phase of the circadian cycle. Similar to previous reports, we performed behavioral tests [68, 69]. After the tests, all equipment was cleaned with 70% ethanol and super hypochlorous water to prevent bias based on olfactory cues. Behavioral tests were performed two tests each day (Fig. 1). It takes 3 h between tests. Mice are back in their home cage in the colony.

### Stress

Animals in the stress groups were subjected to stress once a day according to a protocol similar to that used in previous studies [70–73]. Animals were subjected to stress using the following procedures: (1) tail-pinch for 10 min; (2) forced restraint in a plastic tube for 3 h without access to food or water; (3) hot air (approx. 38 °C) blown using a hair dryer for 10 min; (4) overnight illumination; (5) food and water deprivation for 8 h; (6) damp sawdust (200 mL water absorbed in sawdust bedding). One stressor was applied daily (Fig. 1). Control mice were housed in a separate room, having no contact with the stressed mice.

### General health and neurological screening

Physical characteristics, including body weight, rectal temperature, and presence of whiskers or bald hair patches, were recorded. The righting, whisker twitch, and ear twitch reflexes were also evaluated. Neuromuscular strength was examined using the grip strength and wire hang tests according to a previous study [74]. A grip strength meter was used to assess forelimb grip strength. Mice were lifted and held by the tail so that their forepaws could grasp a wire grid; they then were pulled backward gently until they released the grid. The peak force applied by the mouse forelimbs was recorded in Newtons (N). We performed this test at both P21 and P30.

### Elevated plus maze test

The apparatus consisted of two open arms (8 × 25 cm) and two closed arms of the same size with 30-cm high transparent walls. The arms were constructed of white plastic plates and were elevated to a height of 40 cm above the floor. Arms of the same type were located opposite each other. Each mouse was placed in the central square of the maze, facing one of the closed arms, and was allowed to move freely between the two arms for 10 min. The number of arms entries, distance traveled (m), and percentage of time spent in the open arms were recorded on video and analyzed using video tracking software (ANY-MAZE, Stoelting Co., Wood Dale, IL).

### Social interaction test

The apparatus consisted of a rectangular parallelepiped (30 × 60 × 40 cm). Each mouse was placed in the box for 10 min and allowed to freely explore for habituation. In the sociability test, an unfamiliar C57BL/6N male mouse (stranger mouse) that had no previous contact with the subject mouse was placed into one of the transparent cages (7.5 × 7.5 × 10 cm, which had several holes with a diameter of 1 cm) located at the corners of each lateral compartment. The stranger mouse was enclosed in the transparent cage, which allowed nose contact between the bars but prevented fighting. The subject mouse was placed in the center and allowed to explore the entire box for a 10-min session. One side of the rectangular area was identified as the stranger area and the other as the empty area. The amount of time spent in each area and around each cage during the 10-min sessions was measured. Data were recorded on video and analyzed using the ANY-MAZE software.

### Porsolt forced swim test

The apparatus for the Porsolt forced swim test consisted of four Plexiglas cylinders (20 cm height × 10 cm diameter). The cylinders were filled with water (23 °C) up to a height of 7.5 cm. Mice were placed into the cylinders, and their behavior was recorded over a 6-min test period. In this test, we detect ‘immobile period’ when the animals stop struggling for one second or more. Immobility lasting for less than 1.5 s was not included in the analysis. Data acquisition and analysis were performed automatically using the ANY-MAZE software.

### Tail suspension test

Each mouse was suspended 60 cm above the floor by the tail in a white plastic chamber by an adhesive tape placed < 1 cm from the tip of the tail. Its behavior was recorded for 6 min. Images were captured through a video camera, and immobility was measured. Similar to the Porsolt forced swim test, immobility was evaluated using the ANY-MAZE software.

### Locomotor activity test

For measurements of locomotor activity, the mice were acclimated to the single housing environment for 2.5 h. Locomotor activity data were measured using a photobeam activity system (ACTIMO-100; BRC Co., Nagoya, Aichi, Japan), and activity counts were recorded at 10-min intervals.

### Y-maze test

Spatial working memory was measured using a Y-maze apparatus (arm length: 40 cm, arm bottom width: 3 cm, arm upper width: 10 cm, height of wall: 12 cm). Each subject was placed at the center of the Y-maze field. Visual cues were placed around the maze in the testing room and kept constant throughout the testing sessions. Mice were examined with no learning prior. The number of entries and alterations was recorded and analyzed automatically using the ANY-MAZE software. Data were collected for 10 min [75, 76].

### Statistical analysis of behavioral tests

Data were analyzed with one-way analysis of variance (ANOVA) followed by Tukey’s test, two-way repeated measures ANOVA followed by Fisher’s LSD test, Student’s t test, or paired t test. A *p* value < 0.05 was regarded as statistically significant. Data are shown as box plots.

### Tissue preparation

Following behavioral experiments, we deeply anesthetized the animals with a lethal dose of sodium pentobarbital (120 mg/kg, i.p.) and transcardially perfused them with ice-cold phosphate-buffered saline (PBS) for 2 min and then 4% paraformaldehyde in PBS (pH 7.4) for 10 min (10 mL/min). In all cases, we dissected the brains and post-fixed them overnight with 4% paraformaldehyde in PBS at 4 °C and cryoprotected them by immersion in 15% sucrose for 12 h followed by 30% sucrose for 20 h at 4 °C. To cut sections, we froze the brains in O.C.T. Compound (Tissue-Tek; Sakuma Finetek, Tokyo, Japan) using dry ice-cold normal hexane and we prepared serial coronal sections of 40-µm thickness using a cryostat (CM3050S; Leica Wetzlar, Germany) at − 20 °C. We collected sections in ice-cold PBS containing 0.05% sodium azide.

### Immunohistochemistry

The cryostat sections were treated with 0.1% triton X-100 in PBS for 15 min at 20 °C. After three washes in PBS, the sections were incubated with 10% normal donkey serum (ImmunoBioScience Co., WA) in PBS for 1 h at 20 °C. Sections were again washed three times in PBS and incubated with biotinylated WFA (B-1355, Vector Laboratories, Funakoshi Co., Tokyo, Japan; 1:200) and a primary antibodies in PBS overnight at 4 °C. After washing in PBS, the sections were incubated with streptavidin-conjugated to Alexa Fluor 594 (S11227, Thermo Fisher Scientific, Tokyo, Japan; 1:1000) and secondary antibodies in PBS at 20 °C for 2 h. The sections were rinsed with PBS and mounted onto glass slides using Vectashield mounting medium (H-1400, Vector Laboratories). The prepared slides were stored at 4 °C until imaging.

### Antibodies

The following primary antibodies were used: mouse anti-parvalbumin (clone PARV-19, P3088, Sigma-Aldrich Japan, Tokyo, Japan, 1:1000), mouse anti-NeuN (MAB377, Millipore, 1:1000), rabbit anti-Iba-1 (019-19741, Wako, Osaka, Japan, 1:1000), and mouse anti-Cat-315 (MAB1581, Millipore, Tokyo, Japan, 1:1000). The following secondary antibodies were used: Alexa Fluor 488-conjugated goat anti-mouse IgG (ab150113, Abcam, Tokyo, Japan; 1:1000), FITC-conjugated anti-mouse IgM (sc-2082, Santa Cruz, Texas, USA, 1:1000), and Texas Red-conjugated goat anti-rabbit IgG (TI-1000, Vector laboratories, 1:500).

### Microscopic imaging and quantification of labeled neurons

To quantify the number of PV- WFA-, and Cat-315^+^-positive neurons, confocal laser scanning microscopy of the stained sections was used according to a similar protocol [77]. Images (1024 × 1024 pixels) were saved as TIFF files using the ZEN software (Carl Zeiss Oberkochen, Germany). A 10 ×, or 20 × objective lens and a pinhole setting corresponding to a focal plane thickness of less than 1 μm were used. Data were quantified and presented according to the cortical layer profiles (L2/3 and L5/6) based on fluorescence Nissl staining (NeuroTrace 435/455 blue, N-21479, Molecular Probes, Eugene, OR). All confocal images were converted to TIFF files and analyzed with the Image J software (National Institutes of Health, Bethesda, MD; http://rsb.info.nih.gov/nih-image/). The number of neurons was quantified for at least three coronal sections per animal. The stained neurons or PNNs (defined as neurons with a soma size over 60 μm^2^) were manually tagged and counted within the area of interest. Neuronal density estimates (cells/mm^2^) were also calculated. The data were averaged per mouse. For analyzing PV-, WFA-, and Cat-315^+^-positive PNN morphologies, samples were randomly selected, and high-magnification images using a 100 × objective lens were acquired.

Both fluorescent intensity and soma rea of PV neurons were quantified using at least three coronal sections per animal. Eight-bit grayscale images were captured using a digital camera. The ellipse circumscribing the PV-positive soma and WFA-positive PNNs was traced manually, and the gray levels for PV and WFA labeling were measured using the ImageJ software. We avoided fluorescence saturation by adjusting the exposure time and gain. The same capture conditions were used for all sections. Background intensity was subtracted using the unstained portions of each section. Data are presented as mean ± SEM. The slides were coded and quantified by a blinded independent observer.

### Data analysis of histological quantifications

Data are expressed as the mean ± SEM of five animals per group. Statistical significance was determined by a two-way ANOVA followed by the Bonferroni t test. The statistical significance threshold was set at p < 0.05.

## Results

### Juvenile stress induces changes in body weight gain

**Fig. 2:**
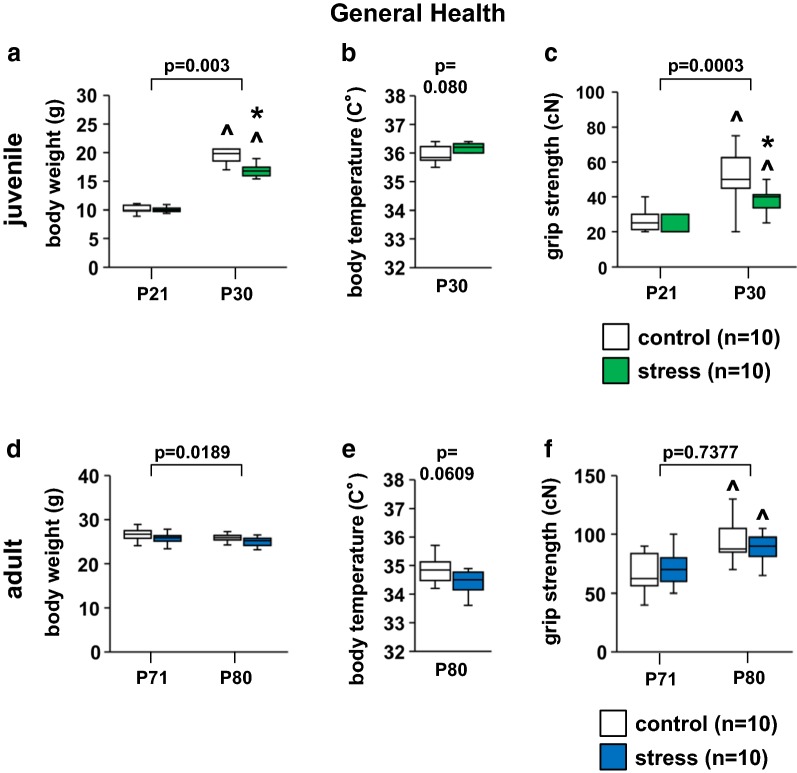
Results of the general health and neurological screening in the stress and control groups. Juvenile: body weight before and after stress (**a**), body temperature (**b**), and grip strength before and after stress (**c**). Adult: body weight before and after stress (**d**), body temperature (**e**), and grip strength before and after stress (**f**). All data are presented as box plots. *Significant difference from control mice (p < 0.05). ^p < 0.05 versus first block; *p < 0.05 between time-matched stress and control groups. The p values were calculated by two-way repeated measures ANOVA (**a**, **c**, **d**, **f**) and Student’s t test (**b**, **e**)

We compared the general health and neurological characteristics of the juvenile-stressed and control groups. We found significant decreases in body weight and grip strength between juvenile-stressed and control group mice on P30 (Fig. 2a, stress × time: *F*_1,32_ = 16.185, p = 0.003; time in control: *F* = 398.249, p < 0.0001; time in stress group: *F* = 219.906, p < 0.0001; stress on P30: *F* = 29.248, p < 0.0001, Fig. 2c, stress × time: *F*_1,32_ = 16.046, p = 0.0003; time in control: *F* = 51.697, p < 0.0001; time in stress group: *F* = 12.371, p = 0.0013; stress on P30: *F* = 21.798, p = 0.0001). There were no significant differences in body temperature between juvenile-stressed and control group mice on P30 (Fig. 2b, *t*_14_ = 2.1448, p = 0.080).

There were no significant differences in body weight, body temperature, and grip strength between adult-stressed and control group mice on P80 (Fig. 2d, stress × time: *F*_1,36_ = 6.046, p = 0.0189; time in control: *F* = 1.303, p = 0.2613; time in stress group: *F* = 1.886, p = 0.1782; stress on P80: *F* = 3.440, p = 0.0719, Fig. 2e, *t*_18_ = 2.1009, p = 0.0609; Fig. 2f, stress × time: *F*_1,36_ = 0.114, p = 0.7377; time in control: *F* = 14.059, p = 0.0006; time in stress group: *F* = 4.183, p = 0.0482; stress on P80: *F* = 1.190, p = 0.2826).

### Juvenile stress did not change anxiety-like behaviors

**Fig. 3:**
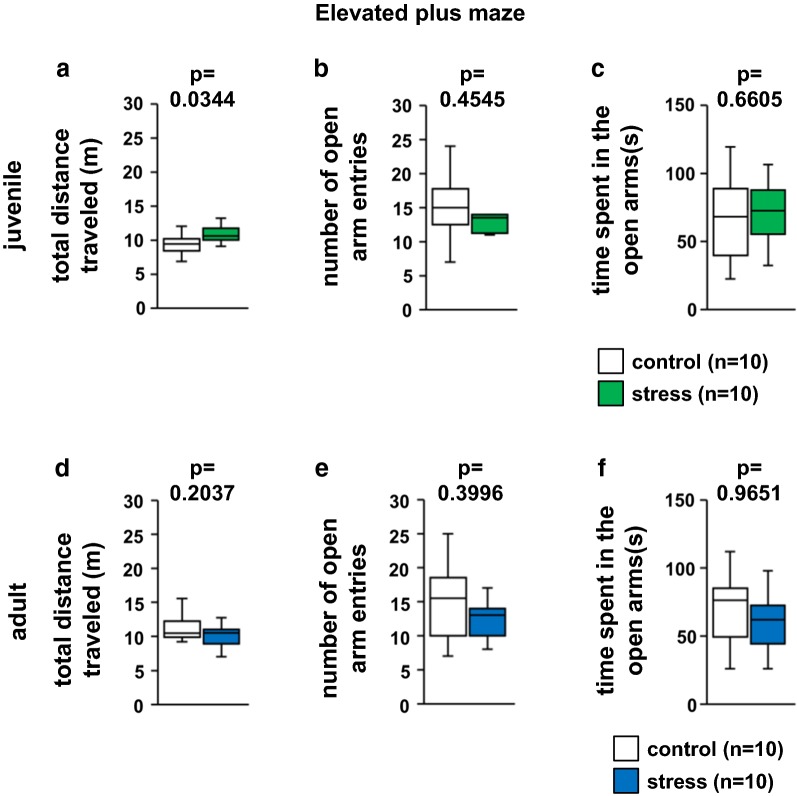
Results of the elevated plus maze test in the stress and control groups. Juvenile: distance traveled (**a**), the number of open arm entries (**b**), and time spent in the open arms (**c**). Adult: distance traveled (**d**), the number of open arm entries (**e**), and time spent in the open arms (**f**). All data are presented as box plots. *Significant difference from control mice (p < 0.05). The p values were calculated by Student’s t test (**a**–**f**)

We evaluated anxiety-like behavior in juvenile stressed mice. In the elevated plus maze test, we observed a significant increase in the total distance traveled in the juvenile-stressed compared with the control group mice (Fig. 3a, *t*_18_ = 2.1009, p = 0.0344). There were no significant differences in the number of total entries into the arms, and the percentage of time spent in the open arms between the juvenile-stressed and control group mice (Fig. 3b, *t*_18_ = 2.1009, p = 0.4545; Fig. 3c, *t*_18_ = 2.1009, p = 0.6605).

Next, we evaluated anxiety-like behavior in adult stressed mice. there were no significant differences in the number of total entries, total distance traveled, and percentage of time spent in the open arms between the adult-stressed and control group mice (Fig. 3d, *t*_18_ = 2.1098, p = 0.2037; Fig. 3e, *t*_18_ = 2.1098, p = 0.3996; Fig. 3f, *t*_18_ = 2.1098, p = 0.9651).

### Juvenile stress reduced depressive-like behaviors

**Fig. 4:**
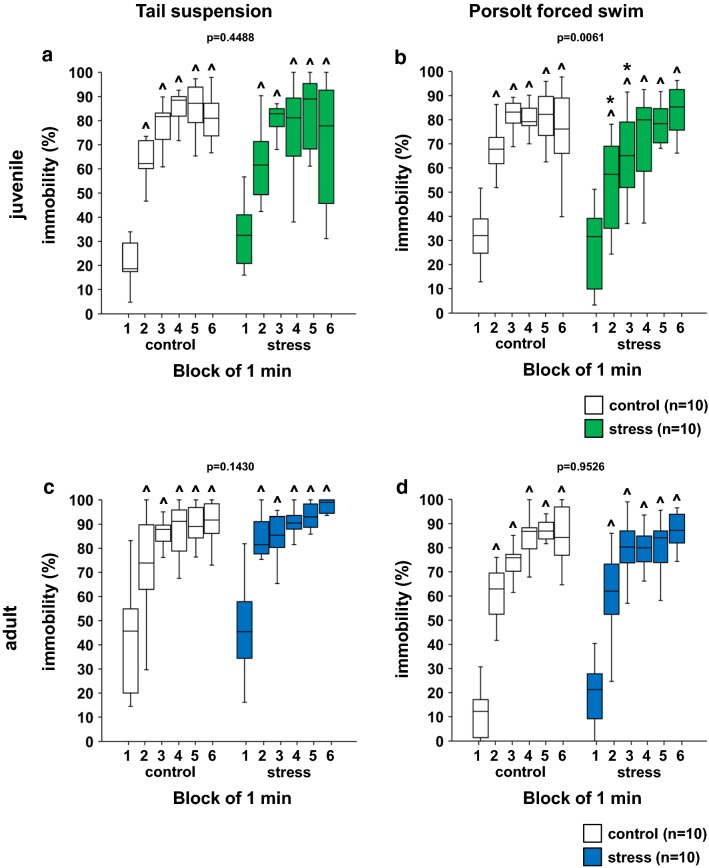
Results of the tail-suspension test and Porsolt forced swim test in the stress and control groups. Juvenile: percentage of immobility time in each 1-min period (**a**) in the tail-suspension test. percentage of immobility time in each 1-min period (**b**) in the Porsolt forced swim test. Adult: percentage of immobility time in each 1-min period (**c**) in the tail-suspension test. Percentage of immobility time in each 1-min period (**d**) in the Porsolt forced swim test. All data are presented as box plots. *Significant difference from control mice (p < 0.05). ^p < 0.05 versus first block; *p < 0.05 between time-matched stress and control groups. The p values were calculated by two-way repeated measures ANOVA (**a**–**d**)

We evaluated depressive-like behavior in juvenile stressed mice. In the tail-suspension test, there were no significant differences in the percentage of immobility time in each 1-min period during the 6-min test period between the juvenile-stressed and control group mice (Fig. 4a, stress × time: *F*_1,102_ = 0.578, p = 0.4488; time in control: *F* = 22.813, p < 0.0001; time in stress group: *F* = 13.792, p < 0.0001). In the Porsolt forced swim test, the juvenile stressed mice spent significantly less time immobile in each 1-min period during the 6-min test period than did the control mice (Fig. 4b, stress × time: *F*_1,108_ = 7.830, p = 0.0061; time in control: *F* = 16.349, p < 0.0001; time in stress group: *F* = 16.883, p < 0.0001).

In the tail-suspension test, we found no significant differences between adult stressed and control mice (Fig. 4c, stress × time: *F*_1,108_ = 2.176, p = 0.1430; time in control: *F* = 66.558, p < 0.0001; time in stress group: *F* = 50.135, p < 0.0001). In the Porsolt forced swim test, during the 6-min test period, there were no significant differences in the percentage of immobility time in each 1-min period in the adult stressed than in the control mice (Fig. 4d, stress × time: *F*_1,108_ = 0.004, p = 0.9526; time in control: *F* = 11.520, p < 0.0001; time in stress group: *F* = 12.582, p < 0.0001).

### Juvenile stress increased activity in a new environment

**Fig. 5:**
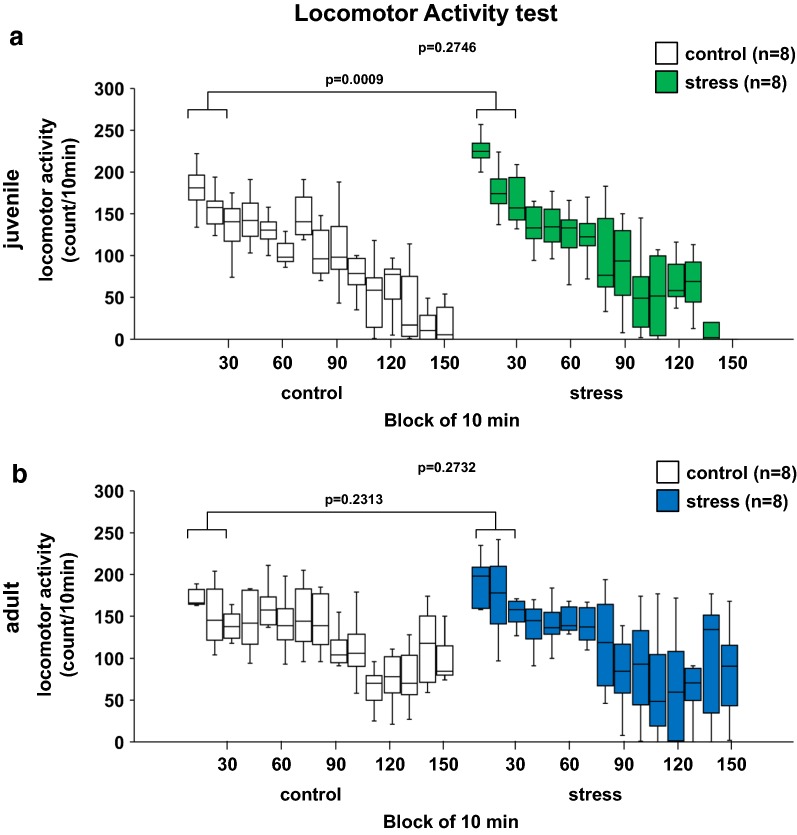
Results of the locomotor activity test in the stress and control groups. Juvenile: spontaneous locomotor activity in each 10-min period (**a**). Adult: spontaneous locomotor activity in each 10-min period (**b**). All data are presented as box plots. *Significant difference from control mice (p < 0.05). The p values were calculated by two-way repeated measures ANOVA (**a**, **b**)

There were no significant differences in locomotor activity during the 150-min period between the adult juvenile stressed and control mice (Fig. 5a, *F*_1,210_ = 1.200, p = 0.2746; Fig. 5b, *F*_1,210_ = 1.207, p = 0.2732). For the first 30-min, we observed a significant increase in the locomotor activity in the juvenile-stressed compared with the control group mice (Fig. 5a, for the first 30-min; stress effect, *F*_1,42_ = 12.750, p = 0.0009; Fig. 5b, for the first 30-min; stress effect, *F*_1,42_ = 1.476, p = 0.2313).

### Juvenile stress showed abnormal social behavior

**Fig. 6:**
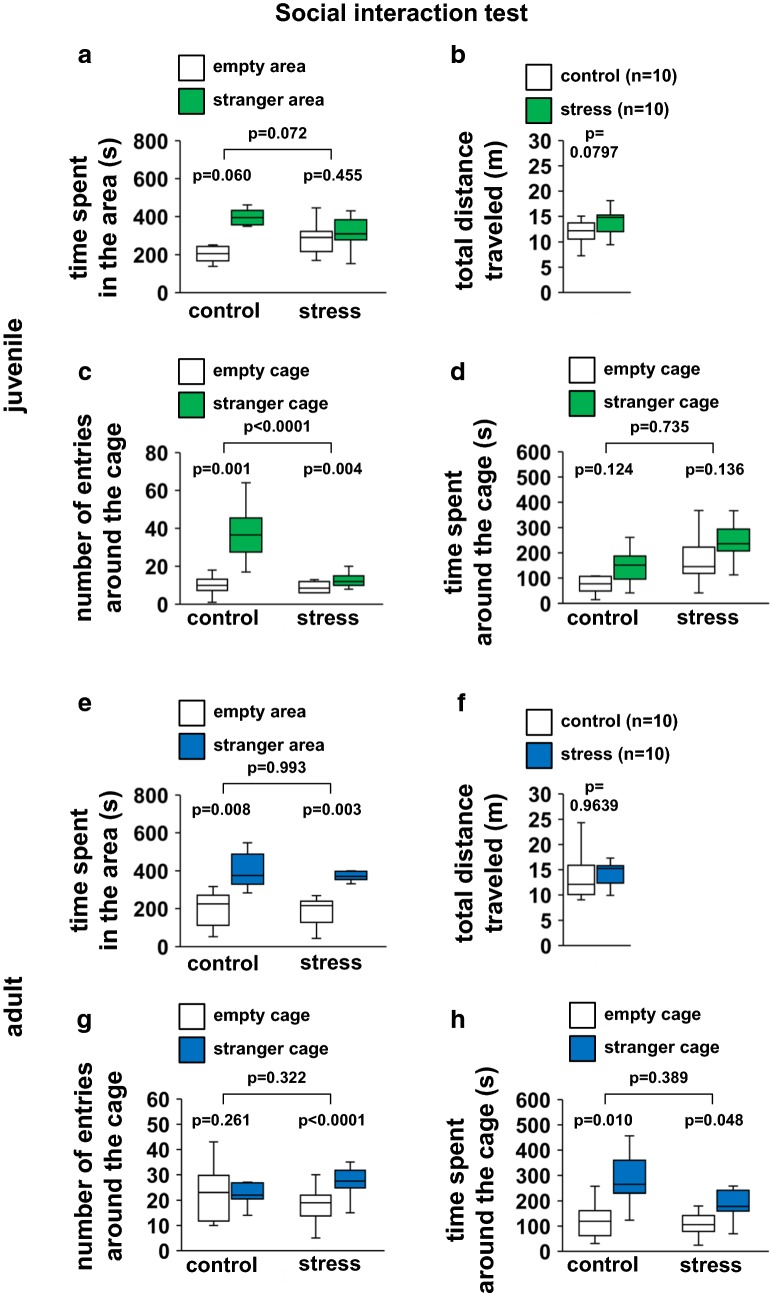
Results of the Social interaction test in the stress and control groups. Juvenile: time spent in the area (**a**), total distance traveled (**b**), number of entries around the cage (**c**), and time spent around the cage (**d**). Adult: time spent in the area (**e**), total distance traveled (**f**), number of entries around the cage (**g**), and time spent around the cage (**h**). All data are presented as box plots. *Significant difference from control mice (p < 0.05). The p values were calculated by two-way ANOVA (**a**, **c**, **d**, **e**, **g**, **h**), one-way ANOVA (**b**, **f**), and paired t test (between the same group in **a**, **c**, **d**, **e**, **g**, **h**)

In the social interaction test, we found several differences between juvenile stressed and control mice (Fig. 6a, *F*_1,36_ = 3.434, p = 0.072; Fig. 6c, *F*_1,36_ = 20.774, p < 0.0001; Fig. 6d, *F*_1,36_ = 0.116, p = 0.735). Control mice spent a significantly longer time in the area containing the novel (stranger) mouse in a transparent cage than in the area containing the empty cage (Fig. 6a, *t*_9_ = −2.147, p = 0.060), but a similar amount of time in both around cages (Fig. 6d, *t*_9_ = −1.695, p = 0.124). Control mice increased the number of entries around the cage containing the stranger mouse than around empty cage (Fig. 6c, *t*_9_ = −4.839, p = 0.001). In contrast, juvenile stressed mice spent a similar amount of time in both areas (Fig. 6a, *t*_9_ = −0.781, p = 0.455), and had similar contact time with both cages (Fig. 6c, *t*_9_ = −3.783, p = 0.004). During the 10-min period, juvenile stressed mice spent significantly more time around the cage containing the stranger mouse than around the empty cage (Fig. 6d, *t*_9_ = −1.638, p = 0.136). There were no significant differences in the total distance traveled between juvenile stressed and control mice (Fig. 6b, *t*_18_ = 2.1009, p = 0.0797). Both control and adult stressed mice spent a significantly longer time in the area containing the stranger mouse than in the area with the empty cage (Fig. 6e, *F*_1,36_ = 0.0001, p = 0.993, empty area versus stranger area: control, *t*_9_ = −3.408, p = 0.008, stress, *t*_9_ = −3.950, p = 0.003), and spent significantly more time around the cage containing the stranger mouse than around the empty cage (Fig. 6h, *F*_1,36_ = 0.760, p = 0.389, empty area versus stranger area: control, *t*_9_ = −3.244, p = 0.010, stress, *t*_9_ = −2.281, p = 0.048). Control mice had a similar number of contacts with both cages (Fig. 6g, *F*_1,36_ = 1.010, p = 0.322, empty area versus stranger area: control, *t*_9_ = −1.200, p = 0.261). Adult stressed mice had an increased number of entries around the cage containing the stranger mouse than around the empty cage (Fig. 6g, *t*_9_ = −5.485, p < 0.0001). During the 10-min period, there were no significant differences in the total distance traveled between adult stressed and control mice (Fig. 6f, *t*_18_ = 2.1009, p = 0.9639).

### Juvenile stress did not change short-term spatial working memory

**Fig. 7:**
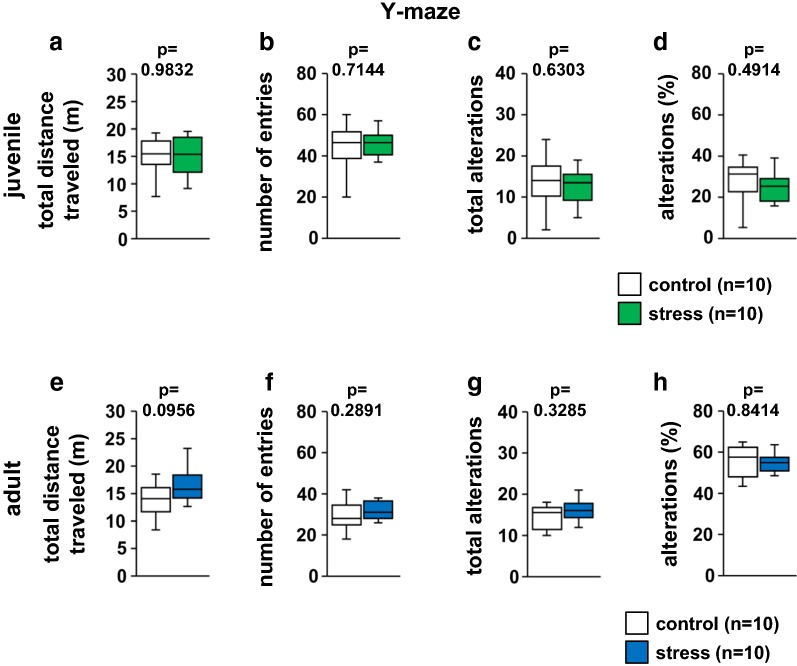
Results of the Y-maze test in the stress and control groups. Juvenile: total distance traveled (**a**), total number of arm entries (**b**), total alternation (**c**), and percentage of alternation (**d**). Adult: total distance traveled (**e**), total number of arm entries (**f**), total alternation (**g**), and percentage of alternation (**h**). All data are presented as box plots. *Significant difference from control mice (p < 0.05). The p values were calculated by Student’s t test (**a**–**h**)

Short-term spatial working memory was examined by monitoring spontaneous alternation behavior in a Y-maze. There were no significant differences in these measures between the juvenile-stressed and control groups in the number of arm entries (Fig. 7b, *t*_18_ = 2.1009, p = 0.7144), in total alternations (Fig. 7c, *t*_18_ = 2.1009, p = 0.6303), alternation percentage (Fig. 7d, *t*_18_ = 2.1009, p = 0.4914), or total distance (Fig. 7a, *t*_18_ = 2.1009, p = 0.9832), indicating that juvenile stress did not affect short-term memory. The results for adult mice were similar (Fig. 7e, *t*_18_ = 2.1009, p = 0.0956; Fig. 7f, *t*_18_ = 2.1009, p = 0.2891; Fig. 7g, *t*_18_ = 2.1009, p = 0.3285; Fig. 7h, *t*_18_ = 2.1009, p = 0.8414).

### Juvenile stress did not change the number of WFA-positive PNNs and PV neurons

**Fig. 8:**
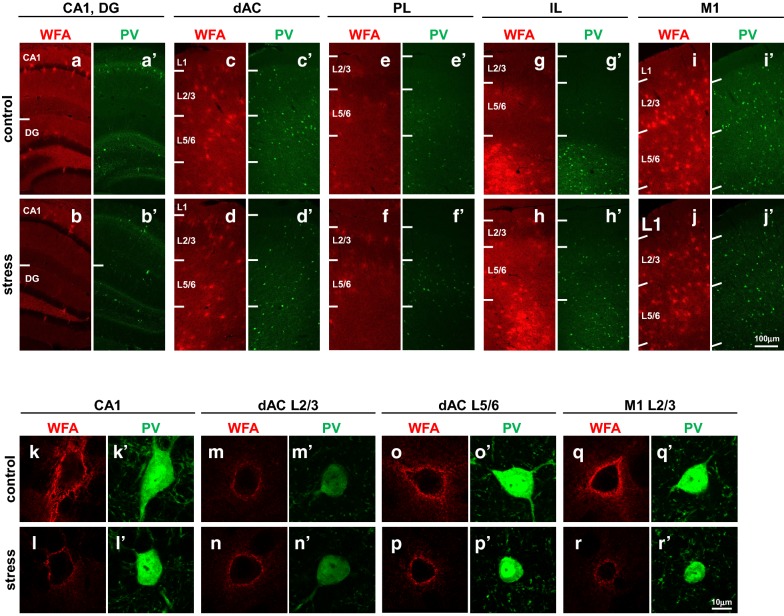
Immunohistochemical detection of PV neurons and WFA-positive PNNs in specific regions of juvenile–stressed and control groups. Representative double immunofluorescence images of the CA1, DG (**a**, **a′**, **b**, **b′**), dAC (**c**, **c′**, **d**, **d′**), PL (**e**, **e′**, **f**, **f′**), IL (**g**, **g′**, **h**, **h′**), and M1 (**i**, **i′**, **j**, **j′**) are shown. PV neurons are indicated by green fluorescence (Alexa Fluor 488), and WFA-positive PNNs are indicated by red fluorescence (Alexa Fluor 594). Representative double immunofluorescence images of the CA1 (**k**, **k′**, **l**, **l′**), dAC L2/3 (**m**, **m′**, **n**, **n′**), dAC L5/6 (**o**, **o′**, **p**, **p′**), and M1 L2/3 (**q**, **q′**, **r**, **r′**) are shown at high magnification. Double confocal images of PV and WFA reactivity in control (**a**, **a′**, **c**, **c′**, **e**, **e′**, **g**, **g′**, **i**, **i′**, **k**, **k′**, **m**, **m′**, **o**, **o′**, **q**, **q′**) and stress mice (**b**, **b′**, **d**, **d′**, **f**, **f′**, **h**, **h′**, **j**, **j′**, **l**, **l′**, **n**, **n′**, **p**, **p′**, **r**, **r′**) are shown. Scale bar = 100 μm in **j′** (applies to **a**–**j**, **a′**–**j′**), and 10 μm in **r′** (applies to **k**–**r**, **k′**–**r′**). *PV* parvalbumin, *WFA Wisteria floribunda* agglutinin, *PNN* perineuronal net, *dAC* dorsal anterior cingulate cortex, *PL* prelimbic cortex, *IL* infralimbic cortex, *M1* primary motor cortex

We examined the effect of juvenile stress on the number of PV neurons and WFA-positive PNNs in several brain regions in juvenile stressed and control mice. Both PV neurons and WFA-positive PNNs were observed in all brain regions analyzed in this study (Fig. 8a–j, a′–j′).

**Fig. 9:**
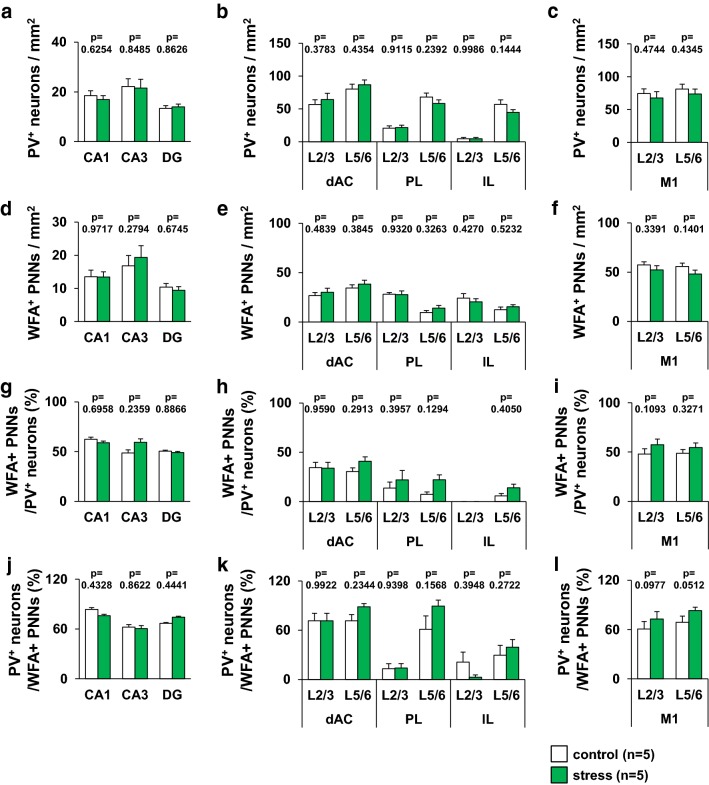
Densities of PV neurons and WFA-positive PNNs in juvenile-stressed or control mice. The region-specific patterns of PV neuron density (**a**–**c**) and WFA-positive PNN density (**d**–**f)** in individual regions are shown. The region-specific pattern of the percentage of PV neurons enveloped by WFA-positive PNNs (**g**–**i**) and the percentage of WFA-positive PNNs that contain PV (**j**–**l**) are shown in the individual regions, hippocampus (CA1, CA3, and DG) (**a**, **d**, **g**, **j**), prefrontal cortex (dAC, PL, and IL) (**b**, **e**, **h**, **k**), motor cortex (**c**, **f**, **i**, **l**) of control or stress mice. All data are presented as the mean ± SEM. *Significant difference from control mice (p < 0.05). The p values indicate two-way ANOVA by Bonferroni *t* test. Abbreviations are the same as those in Fig. 8. **a** Hippocampus; group: *F*_1,66_ = 0.086, region: *F*_2,66_ = 6.449, group × region: *F*_2,66_ = 0.111. CA1: p = 0.6254. CA3: p = 0.8485. DG: p = 0.8626, **b** prefrontal cortex; group: *F*_1,132_ = 0.126, region: *F*_5,132_ = 48.755, group × region: *F*_5,132_ = 0.966. dAC L2/3: p = 0.3783. dAC L5/6: p = 0.4354. PL L2/3: p = 0.9115. PL L5/6: p = 0.2392. IL L2/3: p = 0.9986. IL L5/6: p = 0.1444, **c** M1; group: *F*_1,44_ = 1.140, region: *F*_1,44_ = 0.997, group × region: *F*_1,44_ = 0.002. M1 L2/3: p = 0.4744. M1 L5/6: p = 0.4345, **d** hippocampus; group: *F*_1,66_ = 0.134, region: *F*_2,66_ = 11.937, group × region: *F*_2,66_ = 0.618. CA1: p = 0.09717. CA3: p = 0.2794. DG: p = 0.6745, **e** prefrontal cortex; group: *F*_1,132_ = 0.895, region: *F*_5,132_ = 16.458, group × region: *F*_5,132_ = 0.476. dAC L2/3: p = 0.4839. dAC L5/6: p = 0.3845. PL L2/3: p = 0.9320. PL L5/6: p = 0.3263. IL L2/3: p = 0.4270. IL L5/6: p = 0.5232, **f** M1; group: *F*_1,44_ = 3.048, region: *F*_1,44_ = 0.636, group × region: *F*_1,44_ = 0.144. M1 L2/3: p = 0.3391. M1 L5/6: p = 0.1401, **g** hippocampus; group: *F*_1,66_ = 0.145, region: *F*_2,66_ = 1.550, group × region: *F*_2,66_ = 0.730. CA1: p = 0.6958. CA3: p = 0.2359. DG: p = 0.8866, **h** prefrontal cortex; group: *F*_1,114_ = 0.132, region: *F*_5,114_ = 9.911, group × region: *F*_5,114_ = 6.183. dAC L2/3: p = 0.9590. dAC L5/6: p = 0.2913. PL L2/3: p = 0.3957. PL L5/6: p = 0.1294. IL L5/6: p = 0.4050, **i** M1; group: *F*_1,44_ = 3.446, region: *F*_1,44_ = 0.060, group × region: *F*_1,44_ = 0.6514. M1 L2/3: p = 0.1093. M1 L5/6: p = 0.3271, **j** hippocampus; group: *F*_1,66_ = 0.012, region: *F*_2,66_ = 3.619, group × region: *F*_2,66_ = 0.617. CA1: p = 0.4328. CA3: p = 0.8622. DG: p = 0.4441, **k** prefrontal cortex; group: *F*_1,116_ = 0.886, region: *F*_5,116_ = 17.793, group × region: *F*_5,116_ = 0.905. dAC L2/3: p = 0.9922. dAC L5/6: p = 0.2344. PL L2/3: p = 0.9398. PL L5/6: p = 0.1568. IL L2/3: p = 0.3948. IL L5/6: p = 0.2722, **l** M1; group: *F*_1,44_ = 6.832, region: *F*_1,44_ = 3.364, group × region: *F*_1,44_ = 0.049. M1 L2/3: p = 0.0977. M1 L5/6: p = 0.0512

In all brain regions analyzed in this study, there was no difference in the density of both PV neurons and WFA-positive PNNs between the juvenile stressed and control mice (Fig. 9a–f). There was no difference in the percentage of PV neurons enveloped by WFA-positive PNNs in the hippocampus, prefrontal cortex, and primary motor cortex between the control and the juvenile stressed mice groups (Fig. 9g–i). In all brain regions analyzed in this study, the percentage of WFA-positive PNNs was similar between the juvenile stressed and control mice (Fig. 9j–l).

### Juvenile stress reduces WFA-positive fluorescence intensity but does not change PV-positive fluorescence intensity

An enlarged image of PV neurons and WFA-positive PNNs under the same conditions is shown in Fig. 8, revealing that PV fluorescence intensity and WFA fluorescence intensity differed in each brain region (Fig. 8k–r, k′–r′). In addition, WFA fluorescence intensity differed between the control and juvenile stressed mice.

**Fig. 10:**
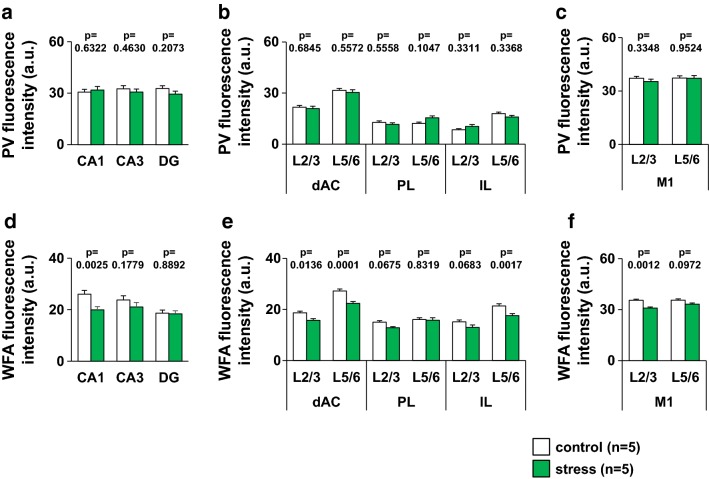
Fluorescence intensity of PV and WFA-positive PNN in each brain region of juvenile-stressed or control mice. Region-specific pattern of the mean fluorescence intensity of PV neurons (**a**–**c**) and WFA-positive PNNs (**d**–**f**) in the hippocampus (CA1, CA3, and DG), prefrontal cortex (dAC, PL, and IL), and motor cortex of control or stress mice. All data are presented as the mean ± SEM. *Significant difference from control mice (p < 0.05). The p values indicate two-way ANOVA by Bonferroni *t* test. Abbreviations are the same as those in Fig. 8. **a** hippocampus; group: *F*_1,210_ = 0.771, region: *F*_2,210_ = 0.052, group × region: *F*_2,210_ = 0.800. CA1: p = 0.6322. CA3: p = 0.4630. DG: p = 0.2073, **b** prefrontal cortex; group: *F*_1,626_ = 0.001, region: *F*_5,626_ = 60.261, group × region: *F*_5,626_ = 1.073. dAC L2/3: p = 0.6845. dAC L5/6: p = 0.5572. PL L2/3: p = 0.5558. PL L5/6: p = 0.1047. IL L2/3: p = 0.3311. IL L5/6: p = 0.3368, **c** M1; group: *F*_1,284_ = 0.526, region: *F*_1,284_ = 0.474, group × region: *F*_1,284_ = 0.411. M1 L2/3: p = 0.3348. M1 L5/6: p = 0.9524, **d** hippocampus; group: *F*_1,210_ = 6.893, region: *F*_2,210_ = 5.811, group × region: *F*_2,210_ = 2.147. CA1: p = 0.0025. CA3: p = 0.1779. DG: p = 0.8892, **e** prefrontal cortex; group: *F*_1,395_ = 30.846, region: *F*_5,395_ = 48.052, group × region: *F*_5,395_ = 1.756. dAC L2/3: p = 0.0136. dAC L5/6: p = 0.0001. PL L2/3: p = 0.0675. PL L5/6: p = 0.8319. IL L2/3: p = 0.0683. IL L5/6: p = 0.0017, **f** M1; group: *F*_1,320_ = 12.119, region: *F*_1,320_ = 1.436, group × region: *F*_1,320_ = 1.274. M1 L2/3: p = 0.0012. M1 L5/6: p = 0.0972

Analysis of fluorescence intensity revealed that both PV and WFA fluorescence intensities differed in each region and each cortical layer of the mouse brain (Fig. 10a–f). There were no differences in PV fluorescence intensity in the hippocampus, prefrontal cortex, and primary motor cortex between control and juvenile stressed mice (Fig. 10a–c). In the CA1 region of the hippocampus, WFA fluorescence intensity was lower in juvenile stressed than in control mice (Fig. 10d). In the dorsal anterior cingulate cortex (dAC) and infralimbic cortex (IL) parts of the prefrontal cortex in juvenile stressed mice, WFA fluorescence intensity was lower than in the same region in control mice (Fig. 10e). In the L2/3 of the primary motor cortex, WFA fluorescence intensity was lower in juvenile stressed than in control mice (Fig. 10f).

### Juvenile stress reduces the soma of PV neurons

**Fig. 11:**
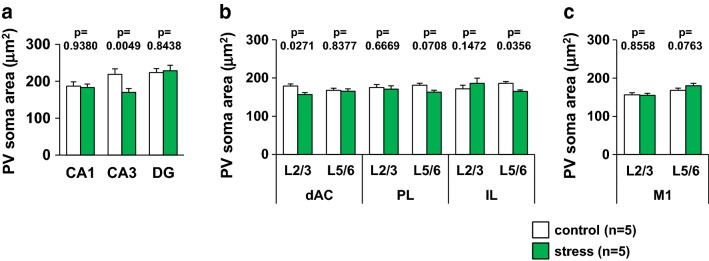
Soma area of PV neurons in each brain region of juvenile-stressed or control mice. Region-specific pattern of the mean soma area of PV neurons (**a**–**c**) in the hippocampus (CA1, CA3, and DG), prefrontal cortex (dAC, PL, and IL), and motor cortex of control or stress mice. All data are presented as the mean ± SEM. *Significant difference from control mice (p < 0.05). The p values indicate two-way ANOVA by Bonferroni *t* test. Abbreviations are the same as those in Fig. 8. **a** hippocampus; group: *F*_1,210_ = 2.477, region: *F*_2,210_ = 4.532, group × region: *F*_2,210_ = 2.832. CA1: p = 0.9380. CA3: p = 0.0049. DG: p = 0.8438, **b** prefrontal cortex; group: *F*_1,626_ = 4.710, region: *F*_5,626_ = 0.842, group × region: *F*_5,626_ = 2.049. dAC L2/3: p = 0.0271. dAC L5/6: p = 0.8377. PL L2/3: p = 0.6669. PL L5/6: p = 0.0708. IL L2/3: p = 0.1472. IL L5/6: p = 0.0356, **c** M1; group: *F*_1,284_ = 1.276, region: *F*_1,284_ = 15.039, group × region: *F*_1,284_ = 1.923. M1 L2/3: p = 0.8558. M1 L5/6: p = 0.0763

We also analyzed the soma of PV neurons in several brain regions of the juvenile stressed and control mice (Fig. 11). We analyzed 575 cells (dAC: L2/3 = 72; L5/6 = 72; PL: L2/3 = 40, L5/6 = 57; IL: L2/3 = 17, L5/6 = 65; M1: L2/3 = 71, L5/6 = 73; CA = 36; CA3 = 36; DG = 36) from control mice, and 567 cells (dAC: L2/3 = 66; L5/6 = 73; PL: L2/3 = 30, L5/6 = 65; IL: L2/3 = 14, L5/6 = 67; M1: L2/3 = 72, L5/6 = 72; CA = 36; CA3 = 36; DG = 36) from juvenile stressed mice. In the CA3 of the hippocampus and L2/3 of the dAC, the area of the soma of PV neurons was smaller in juvenile stressed than in control mice (Fig. 11a, b). No significant differences in soma size were found in the PV neurons of the primary motor cortex between the control and the juvenile stressed mice groups (Fig. 11c).

### Juvenile stress did not change the number of Cat-315-positive PNNs

**Fig. 12:**
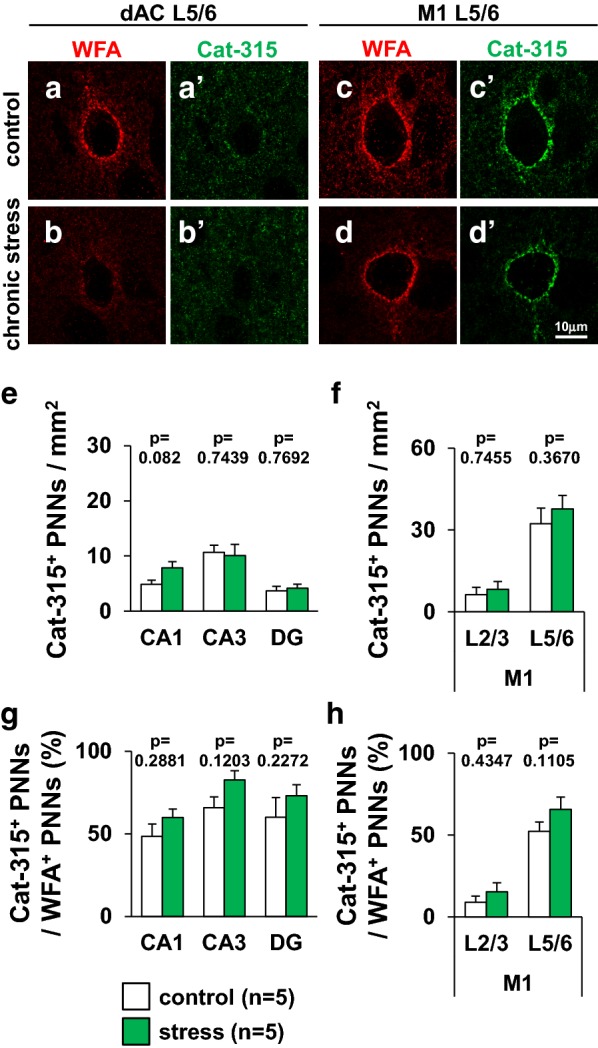
Immunohistochemical detection of Cat-315-positive PNNs and WFA-positive PNNs in specific regions of the juvenile-stressed and control groups. Representative double immunofluorescence images of the dAC L5/6 (**a**, **a′**, **b**, **b′**), and M1 L5/6 (**c**, **c′**, **d**, **d′**) are shown at high magnification. Double confocal images of Cat-315 and WFA reactivity in control (**a**, **a′**, **c**, **c′**) and stress mice (**b**, **b′**, **d**, **d′**) are shown. Cat-315-positive PNNs are indicated by green fluorescence (FITC), and WFA-positive PNNs are indicated by red fluorescence (Alexa Fluor 594). Scale bar = 10 μm in **d**′ (applies to **a**–**d**, **a′**–**d′**). The densities of Cat-315-positive PNN in the hippocampus (CA1, CA3, and DG) (**e**), and primary motor cortex (**f**) are shown. The percentages of Cat-315-positive PNNs co-localized with WFA-positive PNNs in the hippocampus (CA1, CA3, and DG) (**g**), and primary motor cortex (**h**) are shown. All data are presented as the mean ± SEM. *Significant difference from control mice (p < 0.05). The p values indicate two-way ANOVA by Bonferroni *t* test. Abbreviations are the same as those in Fig. 8. **e** Hippocampus; group: *F*_1,54_ = 1.000, region: *F*_2,54_ = 14.729, group × region: *F*_2,54_ = 1.155. CA1: p = 0.0832. CA3: p = 0.7439. DG: p = 0.7692, **f** M1; group: *F*_1,36_ = 0.770, region: *F*_1,36_ = 42.960, group × region: *F*_1,36_ = 0.172. M1 L2/3: p = 0.7455. M1 L5/6: p = 0.3670, **g** hippocampus; group: *F*_1,54_ = 5.000, region: *F*_2,54_ = 0.0339, group × region: *F*_2,54_ = 0.9348. CA1: p = 0.2881. CA3: p = 0.1203. DG: p = 0.2272, **f** M1; group: *F*_1,36_ = 2.943, region: *F*_1,36_ = 66.461, group × region: *F*_1,36_ = 0.5534. M1 L2/3: p = 0.4347. M1 L5/6: p = 0.1105

We examined the effect of juvenile stress on the expression of aggrecan in several brain regions of the juvenile stressed and control mice. The anti-aggrecan antibody Cat-315 is frequently used as a marker of aggrecan-expression on PNNs. We observed more WFA-positive PNNs in the mouse cerebral cortex at P30, while in some brain regions, Cat-315-positive PNN was not yet expressed at P30 (Fig. 12a–d, a′–d′). We did not observe Cat-315-positive PNNs in the mouse prefrontal cortex.

Further, we quantified the number of Cat-315-positive PNNs in the hippocampus and primary motor cortex of the juvenile stressed and control mice (Fig. 12e, f). There was no difference in the density of Cat-315-positive PNNs between juvenile stressed and control mice (Fig. 12e, f). To examine whether juvenile stress affects Cat-315-positive PNN component expression on WFA-positive PNNs in the hippocampus and primary motor cortex of juvenile stressed mice, we quantified the percentage of WFA-positive PNNs co-localized with Cat-315-positive PNNs, and it was similar between the juvenile stressed and control mice (Fig. 12g, h).

### Juvenile stress did not affect immune activation in the central nervous system

**Fig. 13:**
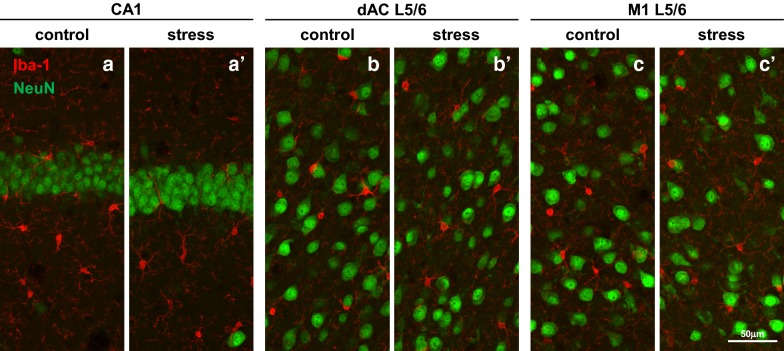
Immunohistochemical detection of Iba-1-positive microglia and NeuN-positive neurons in specific regions of juvenile-stressed and control groups. Representative double immunofluorescence images of the CA1 (**a**, **a′**), dAC L5/6 (**b**, **b′**), and M1 L5/6 (**c**, **c′**) are shown. NeuN-positive neurons are indicated by green fluorescence (Alexa Fluor 488), and Iba-1-positive microglia are indicated by red fluorescence (Alexa Fluor 594). Double confocal images of Iba-1 and NeuN reactivity in control (**a**–**c**) and stress mice (**a′**–**c′**) are shown. Scale bar = 50 μm in **c′** (applies to **a**–**c**, **a’**–**c’**). Abbreviations are the same as those in Fig. 8

To examine whether juvenile stress affects immune activation in the central nervous system of juvenile stressed mice, we observed the morphology of Iba-1-positive microglia in the hippocampus, prefrontal cortex, and primary motor cortex (Fig. 13). Monoclonal antibody Iba-1 is frequently used as a comprehensive marker of microglia. There was no significant difference in the morphology of Iba-1-positive microglia between the control and juvenile stressed mice in the hippocampus, prefrontal cortex, and primary motor cortex (Fig. 13).

## Discussion

In this study, we investigated the influence of stress on behavioral abnormalities and on the development of PV neurons and WFA-positive PNNs in juvenile and adult mice. We discovered that juvenile stress causes increased activity, decreased depressive-like behavior, and social deficits in mice. Furthermore, we revealed that the fluorescence intensity of WFA-positive PNNs decreased in the central nervous system of juvenile stressed mice. These results suggest that juvenile stress affects the development of the mouse brain and causes behavioral abnormalities different from those seen in the case of stressed mature mice.

Juvenile stressed mice had lower body weight and decreased grip strength than control mice. Using the present stress program, mature mice did not show physical changes. Previous studies using juvenile rats and mice also reported that body weight decreases owing to juvenile stress [70, 78]. Decreased essential enzyme secretion for normal cell growth, decreased DNA synthesis, and reduced growth hormone have been implicated as the underlying mechanisms [79, 80]. Indeed, in human studies, it has been reported that the weight of stressed adolescents is lower than that of healthy children [81]. Decreased body weight during the developmental stage may be an indication that the individual is experiencing stress [82].

In the new home cage, juvenile stressed mice showed excessive activity in the first 30 min compared to control mice. In the case of mature mice, such hyperactive behaviors were not observed. In mature mice, the activity level decreases or does not change owing to chronic stress [83–86]. Hyperactivity is a symptom of neuropsychiatric disorders, such as autism spectrum disorder (ASD), and attention deficit hyperactivity disorder (ADHD) [87–90].

Juvenile stressed mice had increased total distance traveled in the elevated plus maze test compared to control mice. An increased total distance seen in the elevated plus maze test may indicate increased locomotor activity or maladaptive-like behavior to the new environment [91]. However, in juvenile stressed mice, increased activity was not observed in all behavioral tests. There were no significant differences in the percentage of time spent in the open arms between the juvenile-stressed and control group mice in the elevated plus maze test. In studies using rats, decreased anxiety-like behavior caused by juvenile stress has been reported [92, 93]. Further studies are needed to elucidate the detailed mechanism.

In this study, depressive-like behavior was decreased in juvenile stressed mice in the Porsolt forced swim test, compared to control mice. Previous studies reported that depressive-like behaviors increase when the animals are exposed to chronic stress [94–98]. In this study, stressed mature mice did not show depressive-like behavior. This result may be due to a shorter stress period than that used in other chronically stressed models [78, 99]. Differences between juvenile stressed and adult stressed mice have also been reported in other studies [78]. It is suggested that these are due to differences in released corticosterone, hypothalamic–pituitary–adrenal depressive-like behavior function, and brain developmental stage [14, 100]. Further studies are needed to elucidate the detailed mechanism; however, based on our results, it is suggested that juvenile mice are more sensitive to stress than adult mice.

In juvenile stressed mice, social preference to stranger mice was reduced compared with control mice. When adult mice were exposed to chronic stress, there was no reduction in sociability [101, 102]. When genetic abnormality occurs in the energy metabolism of PV neurons, social ability is altered in mice [103]. In mice deficient in PV protein, abnormal social behavior and decreased memory ability, such as those seen in ASD-like behavioral abnormalities was observed [46]. In this study, we found WFA-positive PNNs reduced fluorescence intensity around PV neurons in juvenile stressed mice, and it is possible that PV neurons were functioning abnormally. Therefore, in juvenile stressed mice, social preference to stranger mice was altered compared with control mice. Chronic stress affects social interaction and function of paraventricular nucleus [104, 105]. Even in juvenile stressed mice, there may be abnormality in paraventricular nucleus. Further research is necessary to elucidate this mechanism. Abnormal social behavior is a symptom of neuropsychiatric disorders, such as ASD, ADHD, and schizophrenia [106–108].

Adult mice showed an alternation percentage statistically above chance level (50% of alternation) whereas juvenile mice did not in a Y-maze. This indicates that this test is not reliable for juvenile mice.

Juvenile stressed mice showed decreased depressive-like behavior, increased locomotor activity, and abnormal social behavior compared to control mice. It was revealed that chronically stressed juvenile mice showed ADHD- and ASD-like behavioral abnormalities. As described below, there is a possibility that this behavior was caused by PV neuron dysfunction due to decreased PNN condense.

In recent years, it has been shown that the balance between excitation and inhibition in the central nervous system is important for normal brain activity, and any imbalance is believed to cause neuropsychiatric disorder-like behaviors [109, 110]. Abnormalities in the PV neurons have been shown postmortem in the brains of patients with neuropsychiatric disorders, such as schizophrenia and depression [26, 111]. In experimental models, pups born to dams stressed during pregnancy show behavioral abnormalities, indicating decrease in the number of cortical PV neurons and WFA-positive PNNs [112]. The development of PV neurons in the sensory cortex is dependent on sensory inputs, and the development of PV neurons is delayed when sensory inputs are deprived [113–116]. In this study, stress was applied to juvenile mice, but there was no change in both the number of PV neurons and the number of WFA-positive PNNs in the hippocampus, prefrontal cortex, and primary motor cortex. It has been reported that *Vicia villosa* agglutinin (VVA)-positive PNNs increase in the prefrontal cortex of juvenilehood stressed rats within a few weeks [93]. This study showed that stress applied to juvenile mice for approximately 1 week had no obvious influence on the development of PV neurons.

In this study, it was revealed that the fluorescence intensity of WFA-positive PNN in juvenile stressed mice was decreased in the hippocampus, prefrontal cortex, and primary motor cortex compared to control mice. The PNN is a structure enriched with a special extracellular matrix molecule, and a decreased fluorescence intensity is presumed to signify decreased concentration or change in PNN components [115, 116]. Therefore, it is suggested that there was increased synaptic plasticity and increased vulnerability in the brains of juvenile stressed mice compared with control mice. When genetic abnormality occurs in the PNNs formation, social ability is altered in mice [117–119]. It has been reported that stress at a young age changes the hippocampus structurally and functionally [120, 121]. Juvenile stress causes structural and functional changes in the hippocampus [120, 121]. Both the hippocampus and prefrontal cortex are areas vulnerable to stress [62, 122, 123]. In particular, these areas are sensitive to stress during early childhood and adolescence [124, 125]. It has been reported that experiences in early life dramatically alter the structure and function of the brain after maturation [13, 122], and the alterations in PNN extracellular matrix molecules observed in this study could be maintained until maturity [91]. Epidemiological studies have shown that juvenile stress is associated with depression, anxiety, PTSD, and suicide development in adulthood [126, 127]. Chronic stress activates microglia in the central nervous system [128]. However, in this study we have not confirm the activated microglia image. Further studies are needed to elucidate the mechanism of PNN abnormality obtained in this study.

## Conclusions

The present results indicate that juvenile stress affects brain development and causes behavioral abnormalities resembling behaviors linked to developmental disorders in mice. Juvenile individuals are more sensitive and respond differently to stress than mature individuals. The study results may help establish a method to prevent the onset of neuropsychiatric disorders in both juvenilehood and adolescence.

## Abbreviations

ADHD: attention deficit hyperactivity disorder
ASD: autism spectrum disorder
PNN: perineuronal net
PTSD: post-traumatic stress disorder
PV: parvalbumin
WFA: *Wisteria floribunda* agglutinin
